# Risk Analysis of Severe Thrombocytopenia in Nasopharyngeal Carcinoma During Concurrent Radio-Chemotherapy

**DOI:** 10.3389/fonc.2021.754624

**Published:** 2022-02-02

**Authors:** Jialing Hu, Luoyong Tang, Yunqi Cheng, Anwen Liu, Long Huang

**Affiliations:** ^1^ Department of Anesthesiology, The Second Affiliated Hospital of Nanchang University, Nanchang, China; ^2^ Department of Oncology, The Second Affiliated Hospital of Nanchang University, Nanchang, China; ^3^ Jiangxi Key Laboratory of Clinical and Translational Cancer Research, Nanchang, China; ^4^ Queen Mary College, Nanchang University, Nanchang, China

**Keywords:** nasopharyngeal carcinoma, platelet depletion, concurrent radio-chemotherapy, risk factors, predictive factors

## Abstract

**Objective:**

To explore the risk factors and predictive indexes of severe thrombocytopenia during concurrent radio-chemotherapy of nasopharyngeal carcinomas.

**Methods:**

Retrospective analysis was performed from the hospitalized patients with nasopharyngeal carcinoma from August 2014 to July 2017, and induction chemotherapy and concurrent radio-chemotherapy were completed. According to the lowest platelet count during concurrent chemotherapy, patients were divided into observation and control groups. General information and laboratory examinations were recorded and analyzed by univariate analysis, multivariate regression analysis, and ROC curve analysis.

**Results:**

Factors, including age, PLT, IBIL, APTT at first visit, WBC, RBC, HGB, PLT, NEUT, APTT, IBIL, FFA, Crea, and urea before radio-chemotherapy, which are significant in univariate analysis into multivariate regression analysis, were taken. It turned out that RBC (OR = 10.060, 95% CI 2.679–37.777, p = 0.001), PLT (OR = 1.020, 95% CI 1.006–1.034, p = 0.005), and IBIL (OR = 0.710, 95% CI 0.561–0.898, p = 0.004) are independent predictors of severe TP in NPC. ROC analysis showed that the AUC of RBC, IBIL, PLT, and AGE is 0.746 (p < 0.001), 0.735 (p < 0.001), 0.702 (p = 0.001), and 0.734 (p < 0.001). New variables called joint predictors were calculated by a regression equation (Y = 2.309 * RBC - 0.343 * IBIL + 0.02 * PLT - 10.007), the AUC of which is 0.8700 (p < 0.001); best truncation value is >5.87 mmol/l.

**Conclusions:**

Lower RBC, PLT, and higher IBIL before concurrent radio-chemotherapy are independent risk factors causing severe TP during concurrent radio-chemotherapy of NPC. The RBC, PLT, and IBIL before concurrent radio-chemotherapy and joint predictor have a good predictive value to evaluate the risk of severe TP during concurrent radio-chemotherapy of NPC.

## Introduction

Nasopharyngeal carcinoma is a malignant tumor with a high incidence rate and mortality rate in China. The morbidity rate and mortality rate of nasopharyngeal carcinoma in China are 0.019‰ and 0.012‰, which are considerably higher than that over the world (1–3). Its pathogenesis is still unclear, which may relate to genetic factors, environment, infection of EB virus, and so on (4, 5). In clinical cases, the proportion of patients in stages I, II, III, and IV is separately 4.0%, 21.8%, 33.6%, and 32.6%, respectively (6); most patients are in the late stage. Radiotherapy is the principal treatment for nasopharyngeal carcinoma with no distant metastasis. Moreover, it is reported that compared with simple radiotherapy, concurrent radio-chemotherapy can produce an increase of 4%–6% in 5-year overall survival and a decrease of 40%–52% in mortality risk in the patients in T3–4 (6, 7).

As the National Comprehensive Cancer Network (NCCN) suggested, for the patient who is in T1, N1–3, and T2–T4, the therapeutic regimen includes sequential concurrent radio-chemotherapy after induction chemotherapy, and sequential concurrent radio-chemotherapy followed by adjuvant chemotherapy (8). A meta-analysis showed that compared to simple concurrent radio-chemotherapy, applying induction chemotherapy before concurrent radio-chemotherapy can significantly decrease the risk of local development and distal metastasis of nasopharyngeal carcinoma (9). In our department, most patients have been treated with induction chemotherapy plus sequential concurrent radio-chemotherapy (usually docetaxel and platinum) for 3 periods.

Concurrent radio-chemotherapy can increase the survival rate; however, it is also accompanied by the increased risk of complications. Some patients who received induction chemotherapy showed severe platelet depletion, which is a consequence of III~IV bone marrow suppression (BMS) (10), but the platelet count of some other patients appeared to be normal or slightly reduced. The reason of BMS is that chemotherapy can indiscriminately suppress any cells which are actively growing (11); therefore, both tumor and non-tumor cells especially hematopoietic stem cells will be massively killed, leading to BMS. Chemo- and radiotherapy can be acceptable only when the platelet count >80 * 109; when PLT <25*109 which is caused by IV BMS, it can lead to spontaneous hemorrhage, so chemo- and radiotherapy must be stopped or adjusted (12). It is reported that the severe hemotoxic reaction is the main cause of radio-chemotherapy delay (13).

This research is aimed to explore the risk factors and predictive indexes of severe platelet depletion during concurrent radio-chemotherapy of nasopharyngeal carcinomas. Therefore, advanced interference treatment can be available for those patients who might show severe platelet depletion during concurrent radio-chemotherapy. Moreover, there are no similar studies in this field, which brings great insight in the study of chemotherapeutics.

## Methods

### Research Information

All the research objects are the patients from the Department of Oncology, the Second Affiliated Hospital of Nanchang University, from August 2014 to July 2017; information is collected by electronic medical record (EMR). The selection criteria of these patients are the following: (1) pathologically diagnosed as nasopharyngeal carcinoma; (2) induction chemotherapy was performed before concurrent radio-chemotherapy; and (3) the concurrent radio-chemotherapy was completed. Moreover, during concurrent radio-chemotherapy, patients whose lowest PLT ≤ 50 * 10^9^ were placed into the observation group, and whose lowest PLT > 100 * 10^9^ were placed into the control group. The exclusion criteria are as follows: (1) lack of hospitalizing data and (2) incomplete medical records. Each 40 patients were selected into the observation group and the control group according to selection and exclusion criteria.

### Research Method

The general information of patients was recorded in detail, including age, gender, height, weight, PS score, pathological diagnosis, TMN stage, clinical stages, plan and dosage of radiotherapy and chemotherapy, and length of stay during concurrent radio-chemotherapy. Moreover, the laboratory examination of first visit and before radio-chemotherapy was also taken into the record, including a blood routine, coagulation function, D-dimer, liver function, kidney function, blood lipid, C reaction protein, and EBV-DNA. The information of the observation group and control group was organized and analyzed. Univariate and multivariate analyses were proceeded to explore the risk factors and independent predictive factors of severe thrombocytopenia during concurrent radio-chemotherapy, and by using the ROC curve to select the optimal cutoff point and evaluate the predictive value of these risk factors and joint predictor.

### Statistical Analysis

The normality test was used to test continuous variables; the results were described by the mean value standard deviation or median and quartile, then according to the results, T test or non-parametric test was proceeded. Classified variables were described by frequency and constituent ratio and analyzed by the chi-square test. The ROC curve was used to evaluate the predictive value and optimal cutoff point of the predictors, which is suggested by AUC. The independent variables were taken in the binary logistic regression model according to their clinical significance and p < 0.05 in univariate analysis. All the statistical analysis was accomplished by SPSS24 and MedCalc 17.11.5. The significance is determined at p < 0.05.

## Results

### General Information

From August 2014 to July 2017, there are 282 hospitalized patients with nasopharyngeal carcinoma in the Department of Oncology, the Second Affiliated Hospital of Nanchang University; according to the selection criteria, 40 cases were included into the observation group, and 40 control cases were randomly selected, 80 cases in total.

The general information of these patients is shown in **Table 1**; the age between 2 groups (53 vs. 43, p < 0.001) has a significant difference. Age will be included into logistic regression analysis because it is one of the factors that affect the state of bone marrow. Compared with the chemotherapy dosage between 2 groups, the dosage of docetaxel has no significant difference (406.75 vs. 411.50, p = 0.260); the dosage of platinum has a significant difference (562.68 vs. 643.50, p < 0.001). It should be emphasized that the mean dosage of platinum in the observation group is lower than that in the control group. This result indicates that the dosage of platinum is not the factor that causes the different PLT count between 2 groups. Gender, BMI, body surface area, clinical stage, PS scoring, and dosage of radiotherapy between 2 groups have no significant difference (p > 0.05). The baseline information of 2 groups is matched, which is comparable.

**Table 1:** General information.

	Total (N = 80)	Observation group (n = 40)	Control group (n = 40)	P value
Age (years)	49 (16–68)	53 (37–68)	43 (16–66)	**<0.001**
Gender				0.323
Male	56 (70.0%)	31 (77.5%)	26 (65.0%)	
Female	23 (30.0%)	9 (22.5%)	14 (35.0%)	
BMI (kg/m^2^)	23.44 ± 3.87	23.26 ± 3.52	23.61 ± 4.22	0.693
Body surface area (m^2^)	1.66 ± 0.16	1.68 ± 0.18	1.64 ± 0.15	0.262
Clinical stage				
I-II	4	0	4	
III-IV	76	40	36	
ECOG PS				0.723
0	28 (35.0%)	15 (37.5%)	13 (32.5)	
1	44 (55.0%)	23 (57.5%)	22 (52.5%)	
2	8 (10.0%)	3 (5.0%)	5 (12.5)	
Platinum (mg)	603.09 ± 69.21	562.68 ± 72.76	643.50 ± 32.55	**<0.001**
Docetaxel (mg)	406.75 ± 37.38	402.00 ± 36.18	411.50 ± 38.40	0.260
GTVnx(Gy)	70.60 (70.00- 72.2)	71.20 (70.00- 72.2)	70.00 (70.00- 72.20)	0741
GTVnd(Gy)	66.55 (66.00- 68.00)	67.10 (66.00- 68.00)	66.00 (66.00-68.00)	0..870
CTV1(Gy)	64.55 (64.00- 65.80)	65.10 (64.00- 65.80)	64.00 (64.00- 65.80)	0.784
CTV2(Gy)	54.95 (54.40- 56.20)	55.50 (54.40- 56.20)	54.40 (54.40- 56.20)	0.855

Bold values show the significant differences between two groups.

### Comparison of the Laboratory Examinations at First Visit and Before Radio-Chemotherapy Between 2 Groups

Indicators of blood routine analysis, coagulation function, liver function, kidney function, blood lipid level, and creatine kinase at first visit and before radio-chemotherapy were compared. The data at the first visit are shown in **Table 2**. The PLT (190.55 vs. 244.5, p < 0.001), APTT (28.79 vs. 32.50, p = 0.011), and IBIL (8.78 vs. 6.10, p = 0.009) between observation and control groups showed significant differences (p < 0.05); other laboratory results including CRP and WBC showed no significant differences (p > 0.05). The compared data before radio-chemotherapy are shown in **Table 3**; WBC (5.07 vs. 9.61, p = 0.014), RBC (3.57 vs. 4.02, p < 0.001), Hb (108.25 vs. 116.63, p = 0.029), PLT (150.15 vs. 189.15, p < 0.001), ANC (3.15 vs. 7.23, p = 0.018), APTT (27.21 vs. 30.65, p = 0.01), IBIL (7.25 vs. 4.73, p < 0.001), free fatty acid (0.35 vs. 0.23, p = 0.013), creatine (82.53 vs. 68.71, p = 0.01), and urea (5.91 vs. 5.09, p = 0.041) between 2 groups showed significant differences, and other laboratory indicators showed no significant difference (p > 0.05).

**Table 2:** Laboratory examination at first visit.

Laboratory examination	Mean ± Standard deviation		*P*-value
	Observation group	Control group	
CRP (mg/L)	3.62 ± 3.37	6.03 ± 6.68	0.064
WBC (10^9^/L)	6.15 ± 1.71	6.85 ± 2.53	0.150
RBC (10^9^/L)	4.48 ± 0.5	4.59 ± 0.54	0.345
Hemoglobin (g/L)	133.98 ± 13.85	131.53 ± 18.94	0.511
PLT (10^9^/L)	190.55 ± 39.37	244.75 ± 61.81	**＜0.001**
NEU (%)	65.84 ± 8.95	63.83 ± 13.18	0.428
LY (%)	25.82 ± 6.99	25.32 ± 8.5	0.778
MO (%)	6.76 ± 3.44	6.46 ± 2.93	0.681
EO (%)	1.55 ± 1.64	2.45 ± 2.76	0.083
BASO (%)	0.09 ± 0.18	0.09 ± 0.14	1.000
ANC (%)	4.01 ± 1.55	4.33 ± 1.91	0.409
Fibrinogen level (g/L)	2.90 ± 0.95	3.22 ± 0.97	0.154
PT (s)	11.83 ± 2.28	11.47 ± 1.72	0.431
INR	1.00 ± 0.20	0.96 ± 0.14	0.291
PTA (%)	103.91 ± 21.92	112.96 ± 31.99	0.145
APTT (s)	28.79 ± 4.68	32.50 ± 7.69	**0.011**
TT (s)	18.19 ± 2.48	17.99 ± 4.10	0.795
D-dimer (μmoI/L)	0.98 ± 0.88	1.31 ± 2.07	0.363
Total protein (g/L)	70.72 ± 8.58	69.00 ± 5.41	0.290
Albumin (g/L)	41.32 ± 3.92	41.25 ± 2.73	0.923
Globulin (g/L)	29.39 ± 5.6	27.75 ± 3.9	0.134
DBIL (mmol/L)	3.39 ± 1.62	3.90 ± 1.90	0.210
IBIL (mmol/L)	8.78 ± 5.20	6.10 ± 3.51	**0.009**
ALT (U/L)	23.01 ± 12.52	23.74 ± 10.78	0.783
AST (U/L)	30.66 ± 35.09	21.92 ± 5.3	0.123
ALP (U/L)	88.32 ± 2.05	96.49 ± 31.7	0.188
GGT (U/L)	36.49 ± 40.65	31.61 ± 18.02	0.490
CHE (U/L)	7342.16 ± 1903.64	10914.17 ± 16897.63	0.247
TG (mmol/L)	1.25 ± 0.62	1.47 ± 0.82	0.245
PAB (mg/L)	230.82 ± 80.15	236.76 ± 7.88	0.730
TC (mmol/L)	4.36 ± 0.81	4.49 ± 1.09	0.592
FFA (mmol/L)	0.37 ± 0.22	0.37 ± 0.23	0.933
LDL (mmol/L)	2.54 ± 0.71	2.55 ± 0.74	0.968
HDL (mmol/L)	1.19 ± 0.25	1.21 ± 0.28	0.827
LDH (U/L)	182.87 ± 63.78	172.47 ± 40.10	0.449

Bold values show the significant differences between two groups.

**Table 3:** Laboratory examination before radio-chemotherapy.

Laboratory examination	Mean ± Standard deviation		*P*-value
	Observation group	Control group	
CRP (mg/L)	3.62 ± 3.98	3.48 ± 3.48	0.881
WBC (10^9^/L)	5.07 ± 2.22	9.61 ± 11.02	**0.014**
RBC (10^9^/L)	3.57 ± 0.54	4.02 ± 0.50	**＜0.001**
Hemoglobin (g/L)	108.25 ± 15.89	116.63 ± 17.80	**0.029**
PLT (10^9^/L)	150.15 ± 47.79	189.15 ± 54.67	**＜0.001**
NEU (%)	59.51 ± 13.52	63.56 ± 11.68	0.220
LY (%)	30.61 ± 12.84	26.47 ± 13.08	0.157
MO (%)	7.83 ± 4.90	7.67 ± 4.07	0.876
EO (%)	2.01 ± 3.52	2.15 ± 2.50	0.841
BASO (%)	0.038 ± 0.11	0.14 ± 0.38	0.098
ANC (10^9^/L)	3.15 ± 1.95	7.23 ± 10.48	**0.018**
Fibrinogen level (g/L)	3.10 ± 0.88	2.99 ± 0.9	0.628
PT (s)	11.33 ± 1.20	11.44 ± 1.2	0.698
APTT (s)	27.21 ± 5.15	30.65 ± 5.98	**0.010**
TT (s)	17.91 ± 1.78	18.82 ± 4.37	0.254
D-dimer (μmoI/L)	1.25 ± 0.74	1.18 ± 0.52	0.633
Total protein (g/L)	65.55 ± 7.84	66.19 ± 4.99	0.666
Albumin (g/L)	39.76 ± 4.10	40.62 ± 2.8	0.285
Globulin (g/L)	26.33 ± 4.50	25.58 ± 3.12	0.394
DBIL (mmol/L)	2.85 ± 1.55	3.01 ± 1.41	0.631
IBIL (mmol/L)	7.25 ± 3.23	4.73 ± 2.77	**＜0.001**
ALT (U/L)	19.63 ± 9.91	25.21 ± 14.62	0.056
AST (U/L)	22.67 ± 6.57	23.70 ± 6.79	0.502
ALP (U/L)	84.34 ± 25.29	92.42 ± 25.69	0.169
GGT (U/L)	35.03 ± 27.93	37.85 ± 28.92	0.665
CHE (U/L)	7073.73 ± 1766.04	7836.21 ± 1301.36	0.075
TG (mmol/L)	1.78 ± 1.16	345.38 ± 1850.5	0.349
PAB (mg/L)	246.67 ± 1.40	243.91 ± 67.11	0.866
TC (mmol/L)	4.86 ± 0.93	4.75 ± 0.99	0.685
FFA (mmol/L)	0.35 ± 0.26	0.23 ± 0.14	**0.013**
LDL (mmol/L)	2.91 ± 0.8	2.69 ± 0.73	0.292
HDL (mmol/L)	1.170.33	1.17 ± 0.32	0.979
CK (U/L)	68.97 ± 36.25	73.72 ± 9.13	0.594
CK-MB (U/L)	9.83 ± 2.76	9.56 ± 4.14	0.778
LDH (U/L)	158.02 ± 28.61	160.54 ± 28.64	0.746
Creatine (μmoI/L)	82.53 ± 27.24	68.71 ± 7.95	**0.010**
UA (μmoI/L)	353.86 ± 79.7	326.33 ± 91.75	0.166
Urea (mmol/L)	5.91 ± 1.67	5.09 ± 1.77	**0.041**
SOD (U/L)	162.18 ± 27.24	164.15 ± 28.77	0.767

Bold values show the significant differences between two groups.

### Univariate Analysis

In order to explore the risk factor of severe thrombocytopenia during concurrent radio-chemotherapy of nasopharyngeal carcinomas, a binary logistic regression model was established; the change (severe depletion or normal) in PLT count was considered as a dependent variable, and the general information and indicators of laboratory examinations at first visit and before radio-chemotherapy were considered as independent variable. The assignments of observation and control group were 0 and 1. As shown in **Table 4**, univariate analysis found that age (OR = 0.912, 95% CI 0.865–0.963, p = 0.001), PLT (OR = 1.021, 95% CI 1.010–1.033, p < 0.001), IBIL (OR = 0.863, 95% CI 0.767–0.970, p = 0.013) and APTT (OR = 1.126, 95% CI 1.023–1.241, p = 0.016) at first visit, WBC (OR = 1.170, 95% CI 1.014–1.350, p = 0.032), RBC (OR = 5.396, 95% CI 1.970–14.782, p = 0.001), Hb (OR = 1.031, 95% CI 1.002–1.060, p = 0.034), PLT (OR = 1.016, 95% CI 1.006–1.027, p = 0.003), ANC (OR = 1.172, 95% CI 1.0071.364, p = 0.041), APTT (OR = 1.126, 95% CI 1.023–1.241, p = 0.016), IBIL (OR = 0.740, 95% CI 0.614–0.893, p = 0.002), free fatty acid (OR = 0.045, 95% CI 0.030–0.628, p = 0.021), creatine (OR = 0.970, 95% CI 0.945–0.995, p = 0.018), and urea (OR = 0.751, 95% CI 0.565–0.998, p = 0.049) before radio-chemotherapy showed significant differences (p < 0.05).

**Table 4:** Univariate analysis.

Variate	OR value (95% CI)	*p* value
Age	0.912 (0.865–0.963)	0.001
PLT at first visit	1.021 (1.010–1.033)	<0.001
APTT at first visit	1.126 (1.023–1.241)	0.016
IBIL at first visit	0.863 (0.767–0.970	0.013
WBC	1.170 (1.014-1.350)	0.032
RBC	5.396 (1.970–14.782)	0.001
Hemoglobin	1.031 (1.002–1.060)	0.034
PLT	1.016 (1.006–1.027)	0.003
ANC	1.172 (1.007–1.364)	0.041
APTT	1.126 (1.023–1.241)	0.016
IBIL	0.740 (0.614–0.893)	0.002
FAA	0.045 (0.030–0.628)	0.021
Creatine	0.970 (0.945–0.995)	0.018
Urea	0.751 (0.565–0.998)	0.049

### Multivariate Analysis

The above results suggested that laboratory examinations before radio-chemotherapy are more significant and relative. Age and the significant factors before radio-chemotherapy were taken into a binary logistic regression analysis model to proceed multivariate regression analysis. The final regression equation is Y = 2.309 * RBC – 0.343 * IBIL + 0.02 * PLT - 10.007. As shown in **Table 5**, RBC (OR = 10.060, 95% CI 2.679–37.777, p = 0.001), PLT (OR = 1.020, 95% CI 1.006–1.034, p = 0.005), and IBIL (OR = 0.710, 95% CI 0.561–0.898, p = 0.004) are the independent predictors in nasopharyngeal carcinoma during concurrent radio-chemotherapy; low RBC, PLT, and high IBIL are the independent risk factors.

**Table 5:** Multivariate analysis.

	B	Standard error	Ward	DOF	Significance	OR	OR95% CI	
							Lower limit	Upper limit
RBC	2.309	0.675	11.695	1	0.001	10.060	2.679	37.777
PLT	0.020	0.007	8.033	1	0.005	1.020	1.006	1.034
IBIL	-0.343	0.120	8.179	1	0.004	0.710	0.561	0.898
Constant	-10.007	3.101	10.413	1	0.001	0.000		

### ROC Curve Analysis

**Figure 1:**
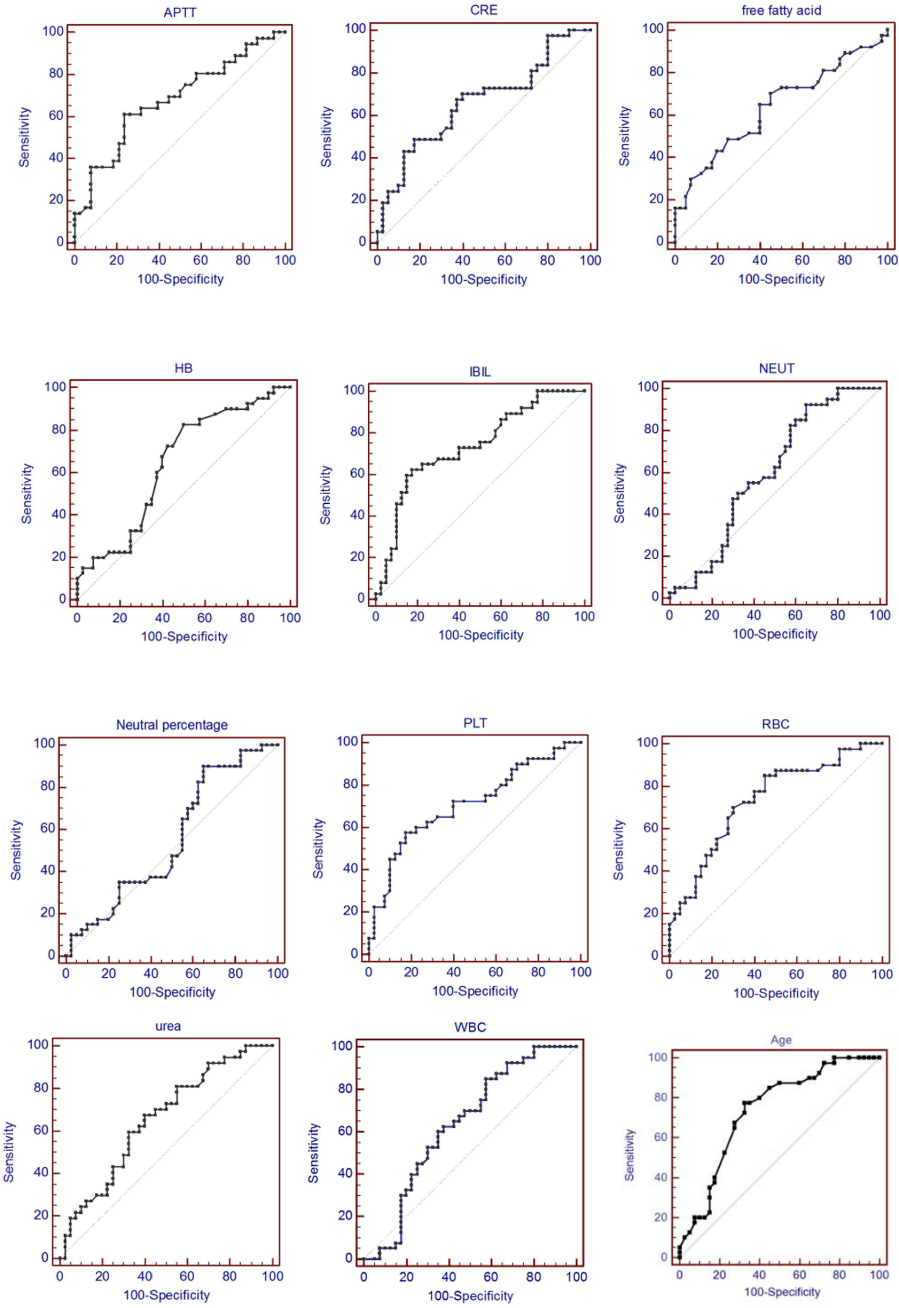
ROC curve of each test variable.

**Figure 2:**
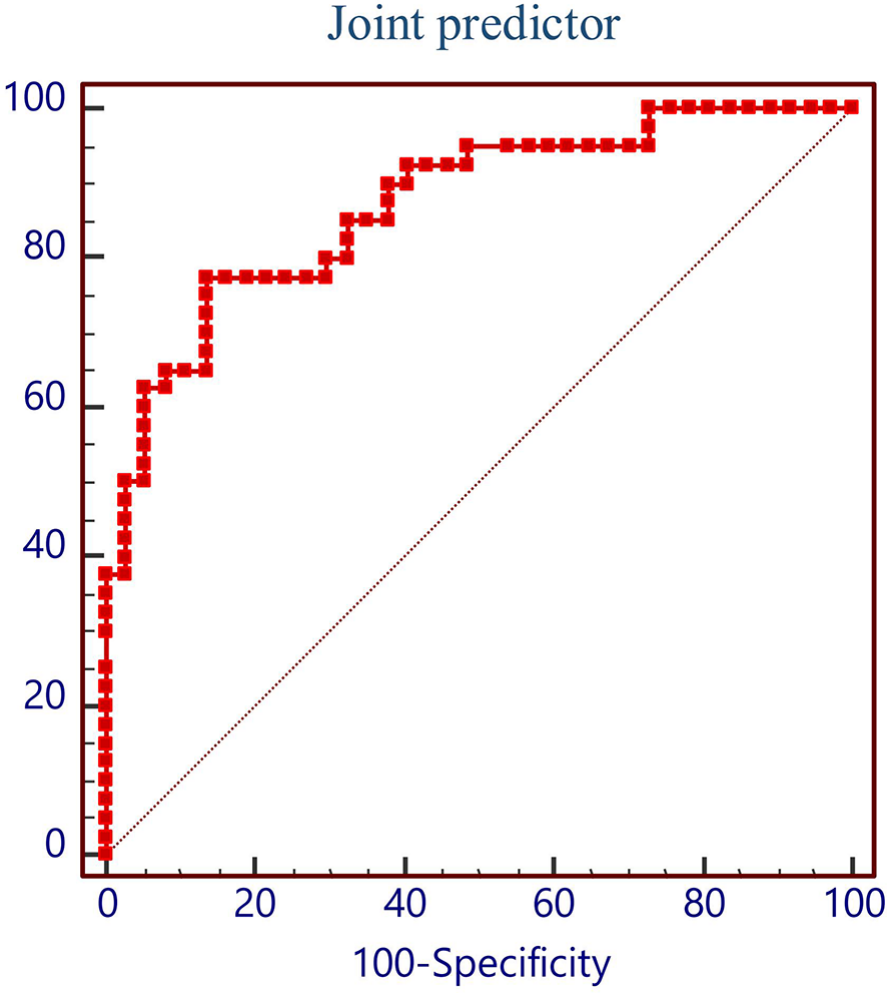
ROC curve of joint predictor.

**Figure 3:**
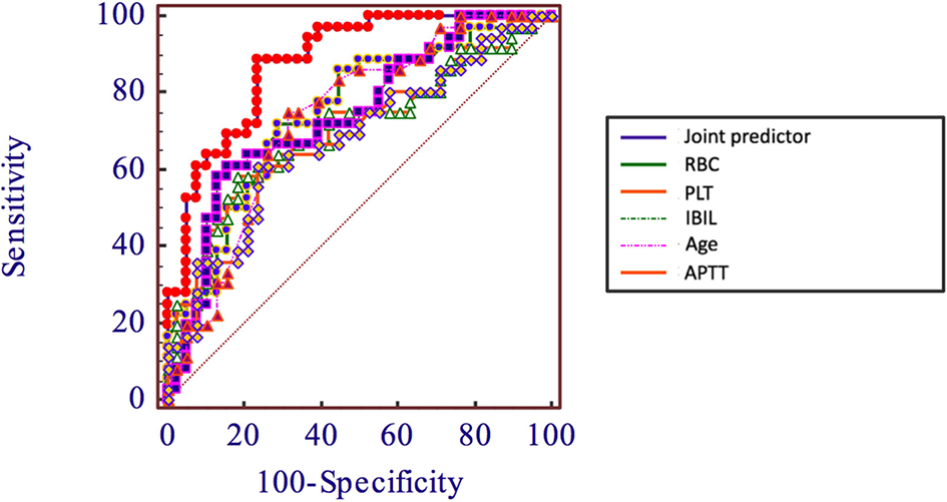
Comparison between joint predictor and other single factors.

In order to evaluate the predictive value of the predictors, the ROC curve (**Figure 1**) was made by considering the significant factors in unifoliate analysis as the test variable. The AUC of these factors is shown in **Table 6**; the sensitivity, specificity, and optimal cutoff point are shown in **Table 7**. Among these, the AUC and optimal cutoff point of RBC are 0.746 (p < 0.001) and ≤3.82, with the sensitivity of 70% and specificity of 70%; the AUC and optimal cutoff point of IBIL are 0.735 (p < 0.001) and >5.87 mmol/l, with the sensitivity of 62.16% and specificity of 82.5%. The AUC and optimal cutoff point of PLT are 0.702 (p = 0.001) and ≤144*109/l, with sensitivity of 57.5% and specificity of 82.5%; the AUC and optimal cutoff points of age are 0.734 (p < 0.001) and >48, respectively, with sensitivity of 72.5% and specificity of 67.5%. We are attempting to evaluate the platelet change during radio-chemotherapy by applying combined diagnosis. As the independent predictors, RBC, PLT, and IBIL of patients can be taken into the regression equation (Y = 2.309 * RBC – 0.343 * IBIL + 0.02 * PLT - 10.007); Y is called joint predictor, and its AUC and optimal cutoff point are 0.870 (p < 0.001) and ≤0.38, respectively, with sensitivity of 86.94% and specificity of 77.50% (**Figure 2**). It has a significant difference (p < 0.05) compared to a single variable, which shows better diagnostic value (**Table 8** and **Figure 3**).

**Table 6:** AUC of each test variable.

Test variable	AUC	SD	95% CI	*p* value
Joint predictor	0.877	0.0484	0.780 to 0.942	<0.001
RBC	0.746	0.057	0.631 to 0.840	<0.001
IBIL	0.735	0.0584	0.620 to 0.831	<0.001
Age	0.734	0.058	0.621 to 0.847	<0.001
PLT	0.702	0.0605	0.585 to 0.803	0.001
APTT	0.679	0.062	0.561 to 0.783	0.008
Urea	0.663	0.0634	0.543 to 0.769	0.020
Creatine	0.660	0.063	0.537 to 0.784	0.015
Hemoglobin	0.643	0.063	0.520 to 0.766	0.028
FAA	0.638	0.064	0.512 to 0.763	0.038
WBC	0.634	0.063	0.510 to 0.758	0.039
ANC	0.600	0.065	0.473 to 0.727	0.122
NEU	0.553	0.066	0.424 to 0.681	0.419

**Table 7:** Predictive value of each test variable.

Test variable	AUC	Sensitivity	Specificity	Optimal cutoff point
Joint predictor	0.877	86.49	77.50	≤0.38
RBC	0.746	70.00	70.00	≤3.82
IBIL	0.735	62.16	82.50	>5.87
Age	0.734	77.50	67.50	>47
PLT	0.708	57.50	82.50	≤144
APTT	0.679	61.11	76.32	≤26.9
Urea	0.663	67.57	60.00	>5.09
Creatine	0.660	48.65	82.50	>80.4
Hemoglobin	0.643	82.50	50.00	≤117
FFA	0.638	70.27	55.00	>0.21
WBC	0.634	85.00	42.50	≤6.43
ANC	0.600	92.50	35.00	≤5.24
NEU	0.553	90.00	35.00	≤73.9

**Table 8:** Comparison of predictive value between joint predictor and single variable.

	Value of area difference	SD	95% CI	Z-statistic	Level of significance
Joint predictor vs. RBC	0.131	0.055	0.023–0.239	2.376	0.018
Joint predictor vs. PLT	0.175	0.061	0.055–0.295	2.868	0.004
Joint predictor vs. IBIL	0.142	0.053	0.038–0.246	2.677	0.007

## Discussion

During clinical practice, the progress of concurrent chemotherapy and even the therapeutic effect are adversely affected due to severe thrombocytopenia. This research explored the risk factors and predictive indexes of severe platelet depletion during concurrent radio-chemotherapy of nasopharyngeal carcinomas. The results suggested that 17.9% of patients suffered severe thrombocytopenia during concurrent chemotherapy. This research made a comparison between patients with and without severe thrombocytopenia and collected their basic clinical information and the laboratory examinations in the first visit and before chemo-radiotherapy to make correlation analysis. The univariate analysis shows that there are statistically significant differences in age, PLT, and APTT in the first visit, and PLT, WBC, RBC, Hb, ANC, IBIL, APTT, FFA, urea, and creatine before chemo-radiotherapy. The laboratory examinations before chemo-radiotherapy are closer in time to chemo-radiotherapy, which have higher significance and research value. Therefore, the results of laboratory examinations before chemo-radiotherapy and age were considered into the multi-factor analysis, which shows that RBC, PLT, and IBIL before radio-chemotherapy are independent risk factors. Then by using ROC regression analysis to evaluate their predictive value, a new variable called joint predictor with a fine reference value was figured out by placing these 3 factors into the regression equation, whose sensibility and specificity are 86.49% and 77.5%, respectively. During clinical practice, whether patients will appear severe thrombocytopenia during concurrent radio-chemotherapy can be predicted by computing the combined predictive factor, and advanced intervention treatment can be conducted.

Thrombocytopenia is a common disease with multiple causes. Aplastic anemia (AA), immune thrombocytopenic purpura (ITP), and thrombotic thrombocytopenic purpura (TTP) are some of the causes of low platelet counts (14). Akin et al. used ITP Bleeding Scale Assessment (ITP-BSA), a bleeding assessment system comprising 11 site-specific grades (15), to assess the performance of patients with above three disorders. The results showed that patients with AA obtained the highest scores and thus confirmed that ITP-BSA is a helpful tool in all patients with thrombocytopenia without regarding the cause (14). In addition, not only blood system diseases but also non-blood system diseases such as liver diseases, hypersplenism, infection, rheumatic diseases, hypothyroidism, radiotherapy, and medication can cause thrombocytopenia. In the department of oncology, the commonest reason that causes thrombocytopenia is chemo- and radiotherapy-caused BMS; BMS is manifested as decreased peripheral RBC, WBC, PLT, and so on, leading to anemia, hemorrhage, and decreased immunity. It is reported that radiotherapy in the lung and pelvic cavity can easily lead to BMS, because the sternum and pelvis are the main parts of hematopoiesis in the adult. While nasopharyngeal carcinoma belongs to the pate tumor, the extent of BMS is relatively lower. In addition, different kinds of chemotherapy drugs can have different effects on BMS. Paclitaxel, vinorelbine, topotecan, gemcitabine, anthracycline, carboplatin, and methotrexate have relatively more severe suppression on bone marrow, while vincristine, pemetrexed, bleomycin, cisplatin, and asparaginase cause lesser suppression on bone marrow.

Bone marrow is the main hematopoietic organ in adult, including hematopoietic cells and hematopoietic microenvironment (HM); hematopoietic cells are composed of hematopoietic stem cells (HSCs), hematopoietic progenitor cells (HPCs), and precursor cells. HSCs are the primitive hematopoietic cells which have strong self-renewal and self-replicated abilities and differentiate into HPCs. HPCs have a limited self-renewal ability; its proliferation and differentiation can meet the demand of normalhematopoiesis and tackle with hematopoietic crisis. During acute BMS, which is a common consequence of chemo-radiotherapy, HSCs differentiate into HPCs in order to maintain normal hematopoiesis; however, when HSCs are harmed by chemo-radiotherapy, a potent bone marrow damage is created. HM is the living environment of HSCs, but also the key link of hematopoietic regulation (16).

This research found that, by comparing the observation group and the control group, the RBC and PLT before chemo-radiotherapy are lower, which are independent risk factors of thrombocytopenia during chemo-radiotherapy. BMS is a common adverse effect of chemotherapy, peripheral WBC, RBC, and PLT count reflect the reserve function of hematopoiesis. The half-life of granulocytes, PLT, and RBC are 6–8 h, 5–7 days, and 120 days, respectively; therefore, the number of the 3 series decreases at different times, granulocytes decrease first, and the decrease in RBC is generally not obvious (12). After 3 periods of chemo-radiotherapy, the bone marrow of patients with nasopharyngeal carcinoma is in a suppressed status, and the WBC count changes obviously, then the PLT count, and finally the RBC count. The RBC and PLT counts before chemo-radiotherapy reflect the bone marrow-suppressed status in patients, which are in accordance with the research results. The univariate analysis showed that there is a statistical difference in age between 2 groups, but multivariate analysis suggested that age is not an independent risk factor. Research indicated that the hematopoietic hypofunction of bone marrow is positively related to aging (17–19), which is not in accordance with the results; the possible reasons are that this research is a single-center, retrospective research, which has its limitation. Therefore, further introspective research with a larger sample size should be followed up. In addition, as an independent predictive risk factor, there is little correlational research on IBIL. Further research on IBIL is necessary.

Meanwhile, lower RBC, PLT, and higher IBIL before radio-chemotherapy are independent risk factors in severe thrombocytopenia during concurrent radio-chemotherapy. Therefore, whether patients will suffer severe thrombocytopenia during concurrent radio-chemotherapy can be predicted by examining RBC, PLT, IBIL, and joint predictor before radio-chemotherapy. However, this research might produce some deficiencies; small sample size and single-center studies can lead to selection bias. Different study centers may have different definitions of severe thrombocytopenia, and the discrepancy among different detecting instruments may lead to different results. Finally, this research is a retrospective analysis, which needs prospective studies to further confirm, and severe thrombocytopenia may also exist in other kinds of tumor therapy; broader and further studies are still waiting to be proceeded.

## Data Availability Statement

The original contributions presented in the study are included in the article/supplementary material. Further inquiries can be directed to the corresponding author.

## Author Contributions

JH: Collect data and statistical analysis, write and revise papers. LT: Collect data, data summary and statistical analysis. YC: Searching for references, helping to write and revise papers. AL and LH: Review and revise the papers and guidance article writing. All authors contributed to the article and approved the submitted version.

## Funding

This work was supported by the National Natural Science Foundation of China [grant number 81960571, 81960468], Key Research and Development Project of Jiangxi province [grant number 20192ACB70013, 20181ACG70011] and Science and Technology Innovation Outstanding Young Talents Training Program of Jiangxi Province [grant number 20192BCBL23023].

## Conflict of Interest

The authors declare that the research was conducted in the absence of any commercial or financial relationships that could be construed as a potential conflict of interest.

## Publisher’s Note

All claims expressed in this article are solely those of the authors and do not necessarily represent those of their affiliated organizations, or those of the publisher, the editors and the reviewers. Any product that may be evaluated in this article, or claim that may be made by its manufacturer, is not guaranteed or endorsed by the publisher.
